# Histochemical Analysis and Distribution of Digestive Enzymes in the Gastrointestinal System of the European Barracuda *Sphyraena sphyraena* (Linnaeus, 1758)

**DOI:** 10.3390/ani14192798

**Published:** 2024-09-27

**Authors:** Ivana Tlak Gajger, Srebrenka Nejedli, Zvonimir Kozarić, Josipa Vlainić

**Affiliations:** 1 Department for Biology and Pathology of Fish and Bees, Faculty of Veterinary Medicine, University of Zagreb, Heinzelova 55, 10 000 Zagreb, Croatia; 2 Department of Anatomy, Histology and Embryology, Faculty of Veterinary Medicine, University of Zagreb, Heinzelova 55, 10 000 Zagreb, Croatia; snejedli@vef.unizg.hr (S.N.); kozaric@vef.unizg.hr (Z.K.); 3 Division of Molecular Medicine, Ruđer Bošković Institute, Bijenička Cesta 54, 10 000 Zagreb, Croatia; josipa.vlainic@irb.hr

**Keywords:** European barracuda, pyloric caeca, intestine, enzymatic activity

## Abstract

**Simple Summary:**

The European barracuda (*Sphyraena sphyraena*) is a wild fish species that is both commercially and ecologically valuable and is found in the Adriatic Sea. To better understand its distribution and physiology, this study examined the digestive enzymes in various parts of the barracuda’s gastrointestinal system using histochemical analyses. This is the first known study to evaluate the activity of aminopeptidase, alkaline phosphatase, acid phosphatase, and non-specific esterase in the pyloric caeca and the anterior, middle, and posterior segments of the European barracuda’s gastrointestinal tract.

**Abstract:**

In this study, we examined the gastrointestinal tract of the European barracuda (*Sphyraena sphyraena*) living in the Adriatic Sea near Dubrovnik, Croatia. The study aimed to identify the presence and distribution of four digestive enzymes: alkaline phosphatase, aminopeptidase, acid phosphatase, and non-specific esterase. We found that alkaline phosphatase activity was present in the brush border of the enterocytes in all the investigated intestinal segments. The activity of the alkaline phosphatase was the strongest in the pyloric caeca but strong only in the basal part of the intestinal villi in the anterior and middle intestinal segments. In the posterior intestinal segment, alkaline phosphatase had strong activity along the entire villi. The activity of acid phosphatase was weak in all the investigated parts of the intestine, except in the posterior part, where it was moderate. Aminopeptidase was detected in the brush border of the intestinal epithelium, with stronger activity in the pyloric caeca and anterior part of the intestine and weaker activity posteriorly. The activity of the non-specific esterase was moderate in the pyloric caeca and anterior part of the intestine, while it was weak in the posterior segment and the lamina propria in all parts of the digestive tract. Weak acid phosphatase activity was observed only in the lamina propria of the posterior part of the intestine. This study is the first to evaluate the activity of digestive enzymes in the European barracuda.

## 1. Introduction

Within the family Barracudas (Sphyraenidae), there are 28 different species [1] varying in size from less than 50 cm to 1.8 m in body length and weighing eight kilograms [2]. The European barracuda or Mediterranean barracuda (*Sphyraena sphyraena*) is an elongated pelagic and predatory fish with two separated dorsal fins, a pointed head, a large mouth, and sharp protruding teeth. This species is globally distributed but mainly inhabits the Mediterranean basin [3,4,5]. Barracudas are popular as food for humans and have gained huge fishery importance. Kalogirou et al. (2012) investigated the feeding habits of the European barracuda originating from the eastern Mediterranean Sea. They concluded that the diet of this fish species is based on the fishes (99%), mainly on pelagic and supra-benthic species, and rarely on cephalopods [6].

The fish gastrointestinal tract’s main functions are digestion, absorption, and transportation of the digested nutrients [7]. Changes in the dietary components with relatively broad diets can modulate fish digestive enzyme activities [8]. The utilization of nutrients depends on the digestive enzyme activity in the proper micro locations along the lumen of the gastrointestinal tract [9,10,11,12,13,14], and it could vary with feeding habits [15], taxonomy [16], pH values [17], and aging [18]. The reproduction success, survival, and growth rates depend on the incoming energy, which is represented by the feeding activity and nutrient quantity and quality. The degree of nutrition digestibility could be explained by understanding the types and functions of digestive enzymes [19,20]. Also, digestive enzymes may be used as an indicator of food acceptance, digestive capacity, and development rate, as well as for survival [21,22]. The digestive enzymes physiology indicates the correlation between their activity and feeding ecology [14].

Previously published study results have described this fish species’ morphology, bite mechanism, predator–prey interactions, length–weight relationships in different microenvironments, behavior and adaptation patterns, and mitogenomic architecture, as well as parasitological examinations and consumers’ risk health assessments with heavy metals linked with fish meat consummation [6,23,24,25,26,27,28,29,30].

To understand fish digestive physiology, histochemical analyses of the gastrointestinal tract are necessary, and currently, for European barracuda, this knowledge gap exists. The present study aimed to determine the histochemical localization and the possible role of four digestive enzymes, namely aminopeptidase (AP), alkaline phosphatase (ALP), acid phosphatase (ACP), and non-specific esterase (NSE), on the intracellular digestion and transport of digested nutrients in the pyloric caeca and anterior, middle, and posterior segments of the intestine in the European barracuda. This study for the first time presents novel information about the nutritional biology of these commercially and ecologically very interesting species, important for their management and conservation in the Adriatic Sea.

## 2. Materials and Methods

**Figure 1:**
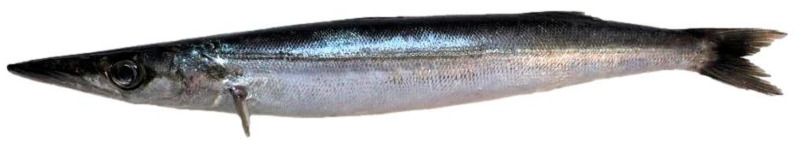
Specimen of examined European barracuda caught with fish for commercial use, intended for human consumption.

This study was carried out on the free-living adult European barracuda (*S. sphyraena* Linnaeus, 1758), caught with fish for commercial purposes during morning hours, near the town of Dubrovnik, in Croatia (Figure 1). The total length (TL) of the investigated fish (n = 12) was from 20.5 to 30.5 cm, and body weight (BW) was from 34 to 75 g.

**Figure 2:**
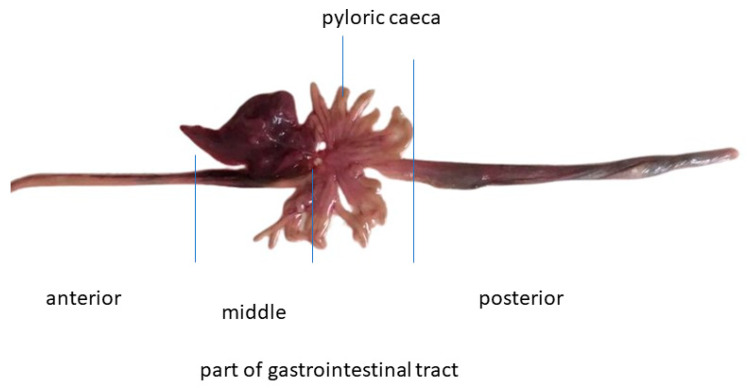
Anatomical macro morphology of investigated features of the gastrointestinal tract of European barracuda.

Immediately after catching, fish were held at a lower temperature on ice, and the dissection was accomplished. Samples of the three segments of the fish specimen intestine (anterior, middle, and posterior) and pyloric caeca were removed (Figure 2) and fixed in cold (4 °C) formol calcium for 24 h and then transferred to an ice-cold sucrose solution. The mentioned tissues were sectioned with a cryocut (Leica Cryocut 1800 Cryostat, Nussloch, Germany) into 10 μm thick slides and used for histochemical detection of enzymatic activity. Histochemical techniques for detecting enzymatic activities were taken from Pearse (1968) [31] and for the AP supstrate-L-Leu-4-MNA (pH 6.5). For the enzymes ALP (E.C.3.1.3.1)-substrate, sodium β-glycerolphosphate (pH 9.4); ACP (E.C.3.1.3.2.)-substrate, sodium β-glycerolphosphate (pH 5.5) and NSE (E.C.3.1.1.)-substrate 1-naphthyl-acetate (pH 6.5), the method according to the Loyda et al. (1979) was used [32]. AP was incubated for 60 min at the temperature of 37 °C. ACP was incubated for 60 min and ALP and NSE for 30 min, at room temperature. After incubation, the sections were rinsed and mounted in glycerin jelly (Canada balsam).

According to the intensity of the color reaction, the enzymatic activity was analyzed visually, using a light microscope (Olympus BX41 phase contrast microscope; Olympus Corporation, Tokyo, Japan) and photographed with an Olympus DP12 U-TVO camera (Olympus Corporation, Tokyo, Japan). All stained sections were examined, with at least ten visual microscopic fields per slide and ten different slides of the same anatomical area, under 10× to 40× magnification. Observations of enzymatic activity were evaluated according to its staining intensity through the adoption of the score ranging from 1 to 4 plus signs and described as very strong reaction (++++), strong reaction (+++), moderate reaction (++), and weak (barely detectable reaction) (+). A score of ++++ was given to those with the highest enzyme activity demand of crucial relevance following the experience of the researcher. Two researchers performed all microscopic observations. To avoid bias, all scores were provided without allowing the view of the scores given by another researcher. The results were calculated from scores received and sorted according to the relevant criteria.

## 3. Results

### 3.1. Morphology and Enzyme Histochemistry

The pyloric caeca and intestine exhibited a uniform histological structure throughout its entire length in all the investigated fish specimens. The mucosa was thrown into many thin folds and contained only two types of cells, columnar epithelial and goblet cells. The numerous goblet cells were distributed across the whole length of the intestine with the lowest number in the pyloric caeca. The major part of the mucosa was formed of columnar epithelial cells. The intestinal layer called tunica muscularis includes the internal circular muscle layer and the external longitudinal muscle bundles. Enzymatic activity in the epithelium of pyloric caeca and all segments of the intestine were related to the columnar epithelial cells.

### 3.2. Aminopeptidase Histochemistry and Distribution

**Figure 3:**
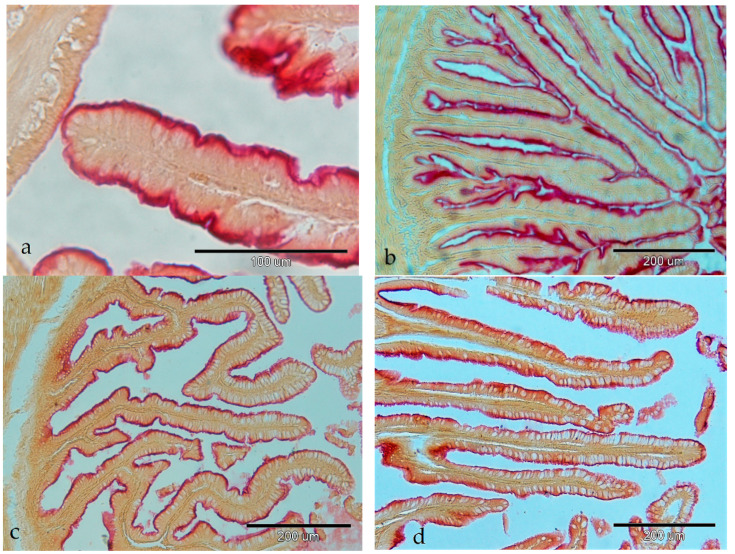
Activity of the aminopeptidase in the gastrointestinal tract of European barracuda: (**a**) pyloric caeca (+++); (**b**) anterior part of the intestine (+++); (**c**) middle part of the intestine (++); (**d**) posterior part of the intestine (+).

Aminopeptidase activity was observed as present in all investigated parts of the gastrointestinal tract. It was visible along the intestinal epithelium brush border. The intensity of AP activity was strong at the top of the absorptive cells in the pyloric caeca (Figure 3a) and in the anterior part of the intestine (Figure 3b). The enzymatic activity of AP was moderately intense in the middle intestine (Figure 3c) and barely detectable in the posterior part of the intestine (Figure 3d).

### 3.3. Alkaline Phosphatase Histochemistry and Distribution

**Figure 4:**
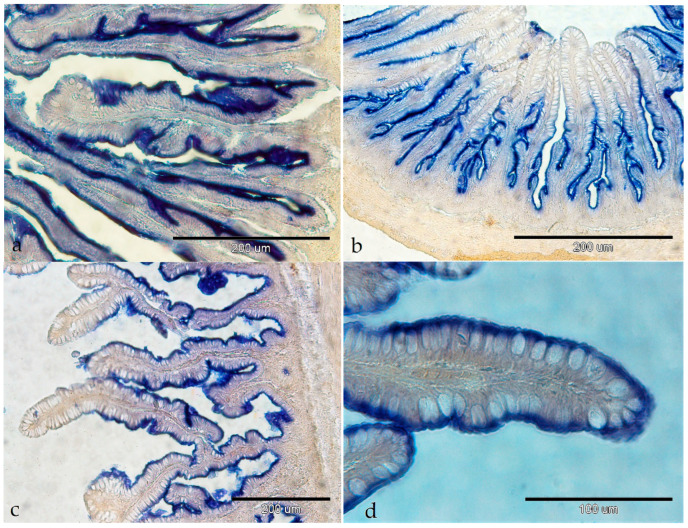
Activity of alkaline phosphatase in the gastrointestinal tract of European barracuda: (**a**) pyloric caeca (++++); (**b**) anterior part of the intestine, base part (1) of the intestine villi (+++); (**c**) middle part of the intestine base part (1) of the intestine villi (+++); (**d**) posterior part of the intestine (+++), goblet cell (+) (2).

Intestinal ALP was detected in the brush border of the enterocytes in all the investigated intestinal segments. This activity was very strong in the pyloric caeca (Figure 4a). In the anterior and middle segments of the intestine, activity was strong just on the basal part of the intestinal villi (Figure 4b,c). In the posterior intestinal segment, ALP activity was strong along the whole villi (Figure 4d) and weak in the goblet cells (Figure 4d).

### 3.4. Acid Phosphatase Histochemistry and Distribution

**Figure 5:**
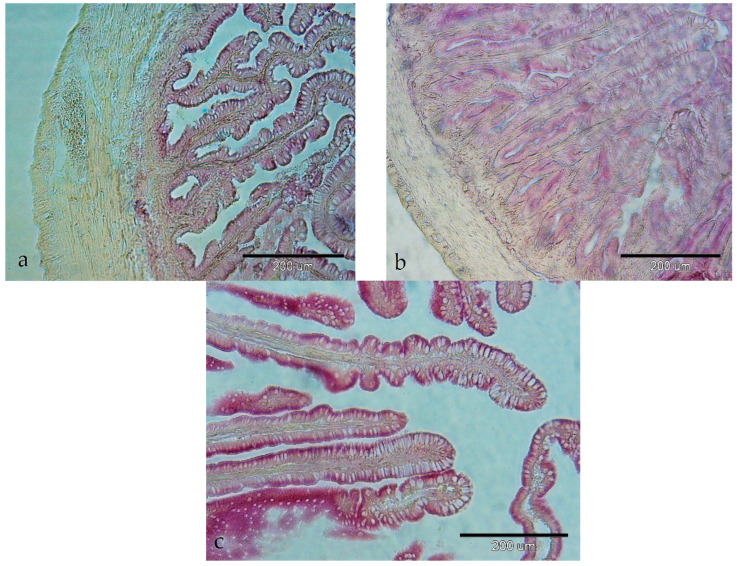
Activity of acid phosphatase in the intestine of European barracuda: (**a**) anterior part (+); (**b**) middle part (+); (**c**) posterior part (++) and in the lamina propria (+).

The activity of ACP was detected as a fine-granular reaction product in the supranuclear region of enterocytes (Figure 5a–c). The activity was weak in all the investigated parts of the digestive tract, except in the posterior part of the intestine, where it was moderate with weak enzyme activity in the lamina propria (Figure 5c).

### 3.5. Non-Specific Esterase Histochemistry and Distribution

**Figure 6:**
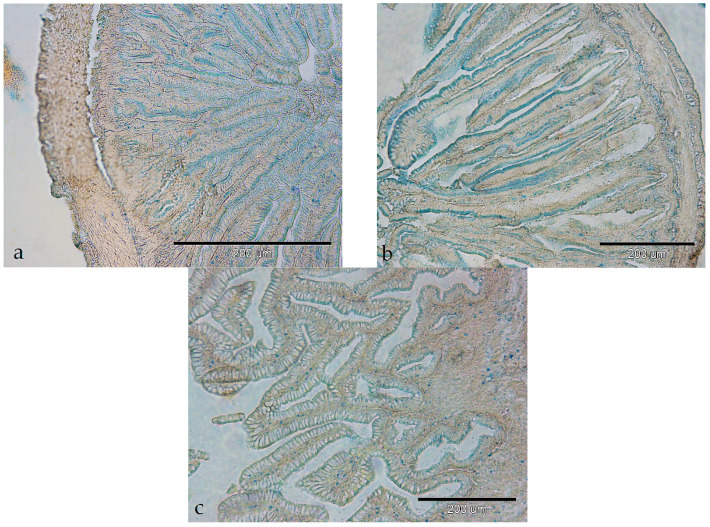
Activity of non-specific esterases in the gastrointestinal tract of European barracuda: (**a**) in pyloric caeca (++), enzyme activity is observed in the lamina propria (+); (**b**) in anterior part of the intestine (++), enzyme activity is observed in the lamina propria (+); (**c**) in posterior part of the intestine (+), enzyme activity is observed in the lamina propria (+).

The activity of NSE was found in the cytoplasm of enterocytes in the pyloric caeca and all the examined intestinal segments. The activity was moderate in the pyloric caeca and the anterior segment of the intestine (Figure 6a,b) with weak activity in the posterior segment (Figure 6c). Weak enzymatic activity was seen in the lamina propria of all the examined parts of the digestive tract (Figure 6a–c).

## 4. Discussion

The utilization of nutrients in the gastrointestinal tract of the European barracuda depends on the fish’s age [18]. The growth rate is highest during the first year of life but decreases later [33]. According to measurements of the body weight and length of the *S. sphyraena* in the investigations of Allam et al. (2004) [34], we could estimate that our investigated fish were between one and two years old.

The pyloric caeca were in the form of multiple finger pouches, which are often present in fish. Through increasing the intestine surface [35], pyloric caeca has an important role in digestion and absorption. Many fish do not have a pyloric caecum. The pyloric caeca and intestine of the *S. sphyraena* showed a uniform histological structure throughout the entire length. The tunica muscularis in the *S. sphyraena* includes the internal circular muscle layer and the external longitudinal muscle bundles.

In our study, goblet cells were distributed along the whole length of the pyloric caeca, and it was previously realized that they secrete mucous for the protection of gut epithelium and enhancement of nutrition substances transportation into the next parts of a gastrointestinal tube [36]. Cho et al. (2023) suggested that a huge number of goblet cells have the function of lubrication in the esophagus of marbled flounder (*Pseudopleuronectes yokohamae*) [37]. Many goblet cells were found throughout all segments of the intestine, like predator fishes such as *S. dumerili* [38]. At the posterior part of the intestine, most herbivorous fishes have a small number of goblet cells [35,39]. In general, mucus substances that are produced by the goblet cells have the function of moistening for facilitating the excretion and protecting the tunica mucosa against parasites, chemicals, and acidity [40,41,42].

Alkaline digestion and nutrient absorption occur in the intestine [43]. From the anterior intestine, the remnants of food particles that were not absorbed migrate into the intermediate and posterior intestines, where the absorption process continues [44]. The possibility to predict the ability of a fish to use different nutrients from the profile of digestive enzymes is suggested by Hofer and Köck (1989) [45]. The intensity and distribution of digestive enzymes vary with intestinal morphology and feeding habits [46]. For digestion, the food must be in direct contact with the digestive enzymes for a certain period. Lower enzyme activity can be compensated with prolonged food retention in the digestive tract [10].

The brush border structure of the marine teleost is linked with the presence of peptidase enzymes, which are important for maximizing the digestion and absorption processes [47]. According to Bates et al. (2007) [48], intestinal AP in zebrafish prevents inflammation caused by the gut microbiota by detoxifying the lipopolysaccharides. Gylfason et al. (2010) found that the intestinal enterocytes of the *Gadus morhua* AP were enriched with lipid raft fractions [49]. The activity of AP in the European barracuda was visible along the intestinal epithelium brush border with strong intensity in the pyloric caeca and the anterior part of the intestine. Moderately intense AP activity was detected in the middle intestine, and it was low in the posterior part of the intestine, suggesting that these segments may also be responsible for peptide digestion. The European barracuda is a predatory fish, and its food is largely made of proteins [6]. The protein content in its diet is probably responsible for the higher activity of AP, an enzyme important for the digestion and absorption of proteins. This enzyme catalyzes the hydrolysis of phosphoric acid monoesters from carbohydrates, fats, and proteins in an alkaline medium and plays a role in the metabolism of calcium, phosphorus, and fatty acids. Also, it participates in regulating pH values in the lumen of the intestine, detoxifying inflammatory components, and modulating the composition of the intestinal microflora [50].

Intestinal ALP is a primary brush border enzyme, and it is important in the absorption of nutrients such as glucose, lipids, inorganic phosphate, and calcium [51]. It also plays many roles in the intestine such as pH regulation, fat acquisition, and anti-inflammatory response, as well as the regulation of the gut microbiome and homeostatic enzymes [52,53,54,55]. ALP contains hydrolase, which is most effective in an alkaline environment [56]. More than half of the reviewed publications reported the highest ALP activity in the anterior part, moderate in the middle, and lowest in the posterior segment of the intestine in both juvenile and older fish [50]. The activity of ALP in European barracuda was strong along all parts of the intestine and very strong in the pyloric caeca. In the anterior and middle segments of the intestine, the activity was strong just on the base of the intestine villi. In these segments, the food probably stands for a longer time than in the posterior intestinal segment in which ALP activity was strong along the whole villi. These results indicate that intracellular protein decomposition occurs along the entire intestine. Higher ALP activity across the intestine has also been reported in farmed *Anguilla anguilla* [12]. In the European barracuda weak activity of ALP was visible in the goblet cells of the posterior part of the intestine which was in accordance with the study which also mentioned ALP activity in the goblet cells of fish [50].

ACP is a lysosomal enzyme that hydrolyzes organic phosphates in an acidic pH [56]. The ACP is one of the marker enzymes for lysosomes, and the activity of this enzyme is related to intracellular digestion [12,13]. Also, it can be linked with pinocytic activity, which could be an alternative pathway for protein digestion [57]. In our study, ACP was detected as a fine granular reaction product in the supranuclear region of enterocytes, which agrees with the previous studies on *Merluccius merluccius* [9], *A. anguilla* [12], *Diplodus vulgaris* [13], and *Cynoglossus semilaevis* [56]. ACP in the European barracuda was weak in all the examined segments of the intestine and moderate in the macrophages of the lamina propria in the posterior segment. In the posterior part of the intestine, ACP activity was also visible in the lamina propria of *Hippoglossus hippoglossus* [58]. Wang et al. (2019) found a similar condition in *Cynoglossus semilaevis*, which coincided with the presence of pinocytotic processes [56]. The presence of proteins within vesicles of the enterocyte’s cytoplasm in the posterior parts of the fish intestine may be an explanation for the stronger enzymatic activity of ACP in these segments [40,59].

NSE is involved in the digestion of glycerol esters of fatty acids [60]. These enzymes may be of particular importance for fish, which can utilize lipids [13]. The intestinal activity of NSE in fishes may influence different feeding styles and environmental conditions [56]. The activity of NSE in the European barracuda was detected in the cytoplasm of enterocytes in the pyloric caeca and in all intestinal segments, which comply with the studies on *Lepidotrigla Cavillone* [11], *Diplodus vulgaris* [13], *Piaractus mesopotamicus* [61], and *Cyprinus carpio* var. koi [62]. The activity of NSE in the European barracuda was moderate in the pyloric caeca and the anterior part of the intestine. In the posterior segment, the activity of the NSE was weak. Similar findings were observed in the wild *A. anguilla* [12]. The weak NSE activity in the posterior part of the intestine leads us to the conclusion that the hydrolysis of carboxylic esters (by non-specific esterase) occurs to a small degree in these parts of the intestine in the European barracuda. The weak activity of the NSE was found in the lamina propria of all the investigated parts of the digestive tract.

In the European barracuda, the final stage of digestion occurs in the enterocytes and depends on the activity of digestive enzymes, which showed segmental variation in the activity. In the pyloric caeca, the activity of ALP was very strong, AP activity was strong, that of NSE was moderate, and ACP activity was weak. The strong activity of the AP and ALP was found in the anterior part of the intestine with weak activity of the ACP and NSE. In the middle segment, the activity of the ALP was strong, AP activity was moderate, and those of NSE and ACP were weak. The activity of the ALP in the posterior segment was strong, the ACP activity was moderate, and those of NSE and AP were weak. Based on the obtained results, we can conclude that the pyloric caeca and all parts of the intestine of the European barracuda are very convenient for the absorption of nutrients such as glucose, lipids, and inorganic phosphate. The digestion and absorption of proteins decrease toward the posterior intestine. In the posterior part of the intestine, the digestion of glycerol esters of fatty acids is a little more intensive than in the other parts of the intestine.

As a recommendation to ensure consistency and reproducibility in the scoring of microscopic slide staining intensity, computer software can be used for quantitative analysis. To the best of our knowledge, this is the first study evaluating the activity of the main digestive enzymes in the described fish species.

## Data Availability

Data are presented in this article.
